# The Political Determinants of Oral Health Inequalities: Lessons in Policy Development and Implementation From Six Case Studies

**DOI:** 10.1111/cdoe.70005

**Published:** 2025-07-27

**Authors:** Stefan T. Serban, Sandra Perdomo, Aida Borges‐Yañez, Finbarr Allen, Carol Guarnizo‐Herreño, María Kamila Navarro‐Ramírez, Matt Hobbs, Lois K. Cohen, Georgios Tsakos, Sarah R. Baker, David I. Conway

**Affiliations:** ^1^ School of Medicine, Dentistry, and Nursing University of Glasgow Glasgow UK; ^2^ Genomic Epidemiology Branch International Agency for Research on Cancer, World Health Organisation Lyon France; ^3^ Facultad de Odontología Universidad Nacional Autónoma de México Ciudad de México Mexico; ^4^ Cork Dental School & Hospital, University College Cork Cork Ireland; ^5^ Facultad de Odontología Universidad Nacional de Colombia Bogotá Colombia; ^6^ Faculty of Health University of Canterbury Christchurch New Zealand; ^7^ College of Health Wellbeing and Life Sciences, Sheffield Hallam University Sheffield UK; ^8^ Lois K. Cohen and Associates LLC Bethesda Maryland USA; ^9^ Department of Epidemiology and Public Health University College London London UK; ^10^ Lincoln Institute for Rural and Coastal Health University of Lincoln Lincoln UK

**Keywords:** health inequities, health policy, oral health, policy analysis, social determinants of health, structural determinants of health

## Abstract

**Objectives:**

Oral diseases are the most prevalent diseases globally, affecting almost half of the world's population with a disproportionate burden on the most vulnerable groups. Despite growing attention on the social and commercial determinants of health, there is still a largely unexplored area in understanding the political determinants of health and oral health. The aim of this paper is to describe national policy development processes for policies impacting population oral health.

**Methods:**

A multiple case study approach was used to analyse six case studies focused on national policy development processes targeting oral health. Kingdon's Multiple Streams Model was used to examine how problems, policy solutions, and political factors aligned to influence policymaking.

**Results:**

Some of the most common barriers to policy adoption and implementation were misinformation strategies, legal challenges, industry lobbying, ideological opposition to state intervention, and lack of transparency regarding conflicts of interest. Important common facilitators included robust scientific evidence presented in an accessible manner to the appropriate audiences, identification of key decision‐makers, support from parties from across the political spectrum, intersectoral collaboration, and ongoing policy monitoring and evaluation.

**Conclusions:**

This study provides novel insights into how political determinants influence social and commercial determinants of health, demonstrating how political contexts and power dynamics shape national public health policy development processes. Understanding these dynamics is essential for ensuring that evidence‐based public health interventions are politically feasible and resilient to opposition from certain private industry and ideological interests. In a time of growing inequalities, neutrality in the face of structural injustice risks entrenching a status quo that favours those with the greatest influence. To address these problems sustainably, public health practitioners must recognise and engage with the political nature of policymaking.

## 1 Background

Inequalities in oral health both within and between countries are a well‐recognised global public health challenge. Oral diseases are estimated to affect almost half of the world's population, with the greatest burden carried by people from the most vulnerable backgrounds [1, 2].

The importance of preventive approaches and health inequalities has been present in health policy discourse for several decades, as reflected in the WHO Constitution (1948) and the Ottawa Charter for Health Promotion (1986); however, the past two decades have seen a renewed policy emphasis on these issues, particularly in relation to the social and commercial determinants of health [3, 4]. This has also been the case for oral health, the public health community increasingly calling for action on the social and commercial determinants of health [5]. These “upstream” approaches shed light on the original causes of the problems and identify policy changes as key to population health improvement and tackling health inequalities [6, 7, 8].

**FIGURE 1:**
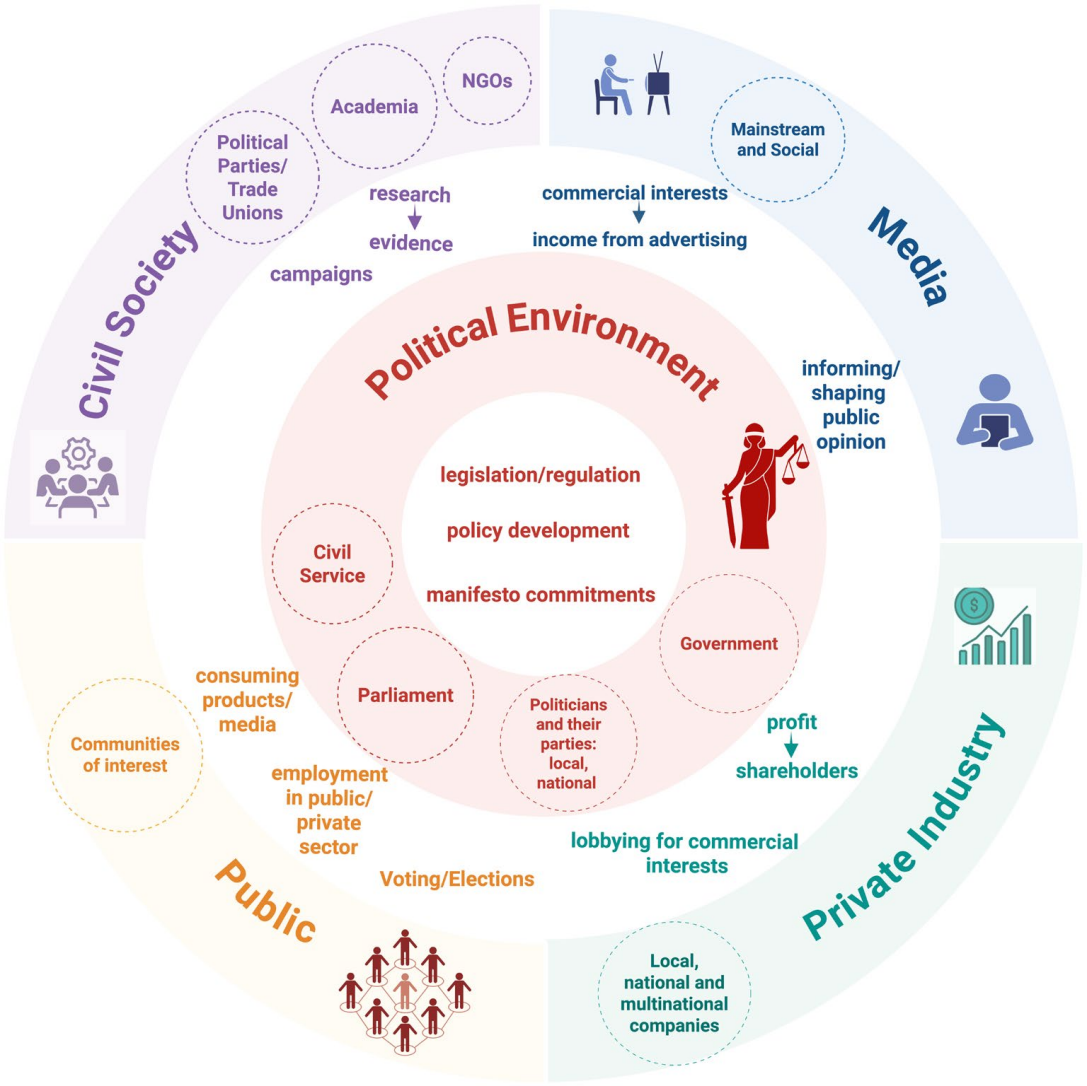
Political determinants of oral health (adapted from Dawes et al. [9]).

The political determinants of health are set out in several frameworks and models such as those proposed by Kickbusch, Dawes and others [9, 10]. Figure 1 presents a summary overview of these models, highlighting the key actors and their actions: the central decision‐making role of governments/parliaments (which could be local, regional and national), where politicians working with government officials/civil servants take political decisions. Politicians are accountable to their constituents and ultimately to the public (the electorate through voting) and typically have party affiliations presenting their political offer through their manifesto commitments. Industry/commercial companies lobby governments in the interest of their shareholders in order to maximise profits. Civic society organisations and individuals (including non‐governmental organisations (NGOs), think‐tanks, trade unions, academia, etc.) can advocate/engage with governments through campaigns and by presenting evidence. Policy decisions also need to take into account financial/economic considerations, security and other competing priorities. The public's relationship with the private industry comes from being consumers of goods and services produced by the industry as well as through employment in various companies. Through elections, politicians are chosen by the public to represent their interests by creating laws, regulations and fiscal measures applicable to both private industry and the population. News outlets and social media are important agents shaping and influencing public opinion, which in turn influences the political agenda [11, 12]. Apart from national governments, there are international organisations which may also influence the development of national policies; there have been a number of significant developments in this area led by the World Health Organisation (WHO), most recently by the adoption of the Bangkok Declaration [13].

To date, most of the scientific literature in relation to the wider determinants of health, has focussed on the social determinants of health, exploring frameworks such as the WHO Commission on Social Determinants of Health and the Dahlgren–Whitehead model [14, 15]. More recently, models of commercial determinants have also been described, with the explicit role of commercial determinants on oral health increasingly recognised [1, 16, 17]. Upstream policy solutions are well described as essential in the efforts to improve population health and oral health, however, the specific means and approaches for developing or changing policy for oral health are less described [18]. Here, we build on these models and articulate the need to go even further upstream, explicitly recognising where the levers of control and power over the social and commercial determinants lie, with the political determinants of health. The political determinants include how “different power constellations, institutions, processes, interests, and ideological positions affect health within different political systems and cultures and at different levels of governance” [19]. These are local/national/global policies, regulations and laws, which may have a *direct* impact on health outcomes—such as through the availability and affordability of healthcare services, healthy food, environment, living and working conditions, or an *indirect* impact through changing the social and economic determinants of health—such as levels of poverty and social security. The distribution of resources across society is ultimately based on political decisions. An example would be the legislative framework, which allows the passing of intergenerational wealth that, in turn, affects socio‐economic status and health outcomes [20]. For most of the history of the United States, property ownership was severely restricted for Black people; therefore, this group started off with a considerable disadvantage in terms of intergenerational wealth, which in turn affected their socioeconomic status and health outcomes [21, 22].

The aim of this paper is to describe national policy development processes for policies impacting population oral health using Kingdon's Multiple Streams Model and to examine how problems, policy solutions and political factors aligned to influence policymaking.

## 2 Methods

We used a multiple case study approach to examine six international case studies of oral health‐related policies [23]. The case studies presented in this paper are primarily explanatory, focussing on the key factors that shaped each policy process, as well as analytical, identifying the broader context relevant to policy development.

The case studies were selected to cover policies on a range of oral health challenges, including child oral health inequalities, the rising prevalence of oral cancers driven by human papillomavirus (HPV) and the common risk factors between poor oral health and certain non‐communicable diseases, such as sugar, alcohol and smoking. Each case study has been reviewed with reference to the related literature, drawing out the key lessons in how the policy was influenced and developed. Additionally, the review of each case study was led by authors based in the country in which the cases originated and who were familiar with the specific policy in order to ensure that the reviews capture the wider cultural and political context in which the policies were developed. This study did not involve a systematic search to assess unavailable documents. The analysis was based on publicly accessible peer‐reviewed publications, policy documents and legislative texts as well as non‐peer‐reviewed publications, including media reports, etc. Given the heterogeneity of sources included in this study, no single standardised quality assessment framework was applicable. Instead, we have taken a pragmatic approach for the inclusion of sources: priority was given to peer‐reviewed publications, official policy documents and legislative texts. Media reports and other non‐peer‐reviewed sources were used selectively, only in instances where official documents were unavailable or where they provided relevant context on the political discourse and public debate. Expert insights from co‐authors familiar with the policy environments were incorporated to contextualise findings as necessary.

The policy processes were analysed using Kingdon's Multiple Streams Model. This robust and widely recognised framework for policy analysis highlights three main factors (streams) required for policy development: the problem, the policy and the politics [24, 25]. The “problem” represents the issue that the “policy” (the solution) needs to address. The “politics” represents the political landscape in which the policy is being developed. An additional element for this model is represented by the “policy entrepreneurs”—influential people who can champion a cause and use their political weight to shape policy development.

Each case study is analysed individually and followed by a summary overview (Table 1) and an evidence synthesis of the shared lessons, barriers and facilitators for policy development, implementation and long‐term continuation.

## 3 Results

### 3.1 Childsmile—The National Child Oral Health Improvement Programme for Scotland

Policy lessons from Childsmile are both in relation to how the programme was established, developed and sustained, but also in relation to the policy development and advocacy work that was integral to the programme.

#### 3.1.1 The Problem

Scotland, in the early 2000s, had among the worst child oral health in Western Europe (nearly 60% of 5‐year‐olds having dental decay) with wide inequalities and no improvement in the previous two decades [26]. Following a national consultation, fluoridation of the public water supply was ruled out due to a large public anti‐fluoridation campaign [27, 28]. Additionally, it was recognised that a traditional health education (message‐based) approach to oral health improvement was both ineffective and could potentially widen inequalities [29]. The subsequent Scottish Government Oral Health Action Plan (2005) identified the need for a national child oral health improvement demonstration programme [30].

#### 3.1.2 The Policy

The Childsmile programme was developed from regional pilot projects [31, 32], which in time were scaled up into a national programme aiming to both improve child oral health and reduce inequalities, and shift the balance of care from treatment to prevention [33].

Since the implementation of Childsmile, substantial improvements in population child oral health have been observed, with dental caries in 5‐year‐old children reducing from 55% in 2003 to 27% in 2024; and in 11‐year‐olds from 47% in 2005 to 18% in 2023; alongside sustained improvements in children from the most socio‐economically deprived communities [34, 35].

The programme is also involved in advocacy for the development of other national policies and regulations relating to diet and nutrition. This included participating in a multidisciplinary working group, which developed government regulations relating to healthy eating in schools [36]. It also takes a common risk factor approach relating to non‐communicable diseases and promotes the integration of oral health into national strategies and policies relating to reducing sugar consumption and obesity [37, 38]. The Childsmile evaluation team has supported the evaluation of the UK Sugar Sweetened Beverage Taxation [39]. The successes of Childsmile have been recognised by the European Commission as a gold standard public health intervention [40].

With Scottish Government support and working in partnership with the National Health Service (NHS) Scotland, the Childsmile programme also directly influenced changing the primary care dentistry payment contract and system in 2011. This enabled dental teams to deliver (and be paid for) more preventive care for children, which was continued into the more recent 2023 dental service reforms [32, 41]. Other regulatory and policy changes include successfully lobbying the General Dental Council (the UK regulator for dental professionals) for a change in the scope of practice of dental nurses. This was to enable appropriately trained dental nurses to apply fluoride varnish in nurseries and schools and in dental practice settings, which had not been part of their scope of practice before [42]. Additionally, a whole new health care workforce role was created as the Childsmile community‐based Dental Health Support Workers [42].

The evaluation of Childsmile is embedded in the programme, academically led, theory‐based, multi‐disciplinary guided by the programme logic models. It provides outcome and process evaluation through population‐level data linkage, community trials, economic evaluations, investigations drawing from behavioural and implementation science, evidence reviews and updates, and applications of systems science. Multiple programme partners are involved in collaborative working to identify what aspects of the programme are working (and should be maintained) and to address areas that may not be working as well, with findings feeding into the development of local and national Childsmile policies [43]. An example of the programme providing early wins for ensuring ongoing government support was evidence of the cost effectiveness of the nursery supervised toothbrushing programme, which indicated that the estimated cost of nursery toothbrushing in Scotland was around £1.8 million per year [44]. The estimated costs associated with the dental treatments for 5‐year‐old children decreased over time, such that eight years into the toothbrushing programme, the expected annual savings were more than two and a half times higher than the costs of the programme implementation (£4.7 million) [45]. This case study of preventive spend was widely used across government health policy [45].

#### 3.1.3 The Politics

Successive Scottish Governments' strong commitment to Childsmile as the cornerstone of national child oral health improvement policy has been a key factor in the programme's success. Since the start of the programme, there has been a succession of governments with varying political compositions: the 2003 Labour (centre‐left)—Liberal Democrat (centrist to centre‐left) coalition, followed by the 2007 minority government led by the Scottish National Party (SNP) (centre‐left). This was succeeded by an SNP majority government in 2011, and SNP‐led minority governments following the 2016 and 2021 elections, the latter supported by a formal cooperation agreement with the Scottish Green Party (centre‐left to left‐wing). Despite these changes, each government continued and built on the programme, which has enabled it to sustain and take a long‐term view rather than be restricted to the lifetime of a parliament. Throughout the development and implementation of the programme, regular and continued engagement and communication with policymakers and stakeholders at all levels was crucial to building and sustaining successful partnerships. This involved engaging with partners on their own terms and in their language. For example, with the education sector, this included engaging at all levels from within Scottish Government between the Ministries of Education and Health, in local government with Directors of Education and Health Board (health authority dental leads), and between local dental services and local headteachers (in nurseries/kindergartens). Communication involved: highlighting the potential of improving oral health and reducing school absences; showing that delivering daily supervised toothbrushing in nurseries was supporting delivery of the education curriculum on health and well‐being and self‐care [46], and co‐developing with education partners detailed toothbrushing guidelines policy [47]. These policy principles have been adapted and adopted or are being adopted in a number of countries across the world including Chile, England, Malawi, the Netherlands, Saudi Arabia and Vanuatu [48].

### 3.2 National Sugar‐Sweetened Beverages Tax in Mexico

The policy lessons from the development of the national sugar‐sweetened beverage (SSB) tax in Mexico highlight the importance of identifying a suitable policy window for action and cross‐sector cooperation between civil society and academia. The strong influence of the sugar industry in policy development suggests the need to scrutinise potential conflicts of interest between politicians and industry.

#### 3.2.1 The Problem

By 2012, in Mexico, 73% of women, 69% of men and 30% of children and adolescents were overweight or obese [49]. National data showed that the overall prevalence of overweight and obesity increased dramatically from 34.5% in 1988 to 61% in 1999 and to 69.3% in 2006, while increases were observed across all age groups [50]. The prevalence of type 2 diabetes and other obesity‐related diseases was also very high, with important socio‐economic consequences [51]. In terms of oral health, the prevalence of caries experience was 77.7% among children and adolescents and 92.6% among adults [52]. High sugar consumption was key to this public health crisis, with 19% of all diabetes, cardiovascular disease and obesity‐related cancer deaths being attributable to the consumption of sugar‐sweetened beverages (SSB) [53]. Mexico had the highest per capita soft drinks consumption in 2010 across 75 countries, and was the second largest consumer of ultra‐processed foods (UPF) and beverages in the Latin American region between 2009 and 2014 [54, 55]. Sugar consumption and its health consequences were higher among poorer population groups and those living in rural areas [56].

For many years, public health initiatives were either based on recommendations and messages about lifestyle individual choices or not enforced enough to ensure their compliance. For example, the 2010 “National Agreement for Food Health” was voluntary and was not supported by laws or regulations to facilitate its implementation.

#### 3.2.2 The Policy

The fiscal reform was brought forward through joint and persistent advocacy efforts from different stakeholders. In May 2012, the book “Obesity in México: Recommendations for a State Policy” presented scientific evidence about the issue and recommended SSB taxation [57]. In December 2012, a legislative proposal was presented regarding the application of a Special Production and Services Tax (Impuesto Especial Sobre Producción y Servicios—IEPS) to SSB, but it had not been discussed by the end of the first legislative year and was therefore automatically rejected. A new government took office in 2013 and sought to increase revenues to the treasury and reduce obesity levels by increasing taxes on all foods, medicines and non‐alcoholic drinks. This measure was too unpopular, but it opened a “policy window” for framing the issue around a “health tax” for advocating for a soda tax. The issue continued to be raised and gain relevance, in both chambers of the Mexican Parliament, as well as the media and NGOs such as Contrapeso, Oxfam, Fundación Mídete, Alliance for Food Health and Consumer Power [58]. In addition, the involvement of the National Institute of Public Health and the Ministry of Health, with the backing of the local office of the Pan‐American Health Organisation provided fundamental political support. The proposal became a joint effort of an intersectoral group including the federal government, congress, academia, NGOs (both local and international) and international health agencies [57, 59, 60].

In 2013, the Mexican Government implemented the “National Strategy to Prevent and Control Obesity and Diabetes”, which included limiting food marketing exposure to children, implementing front‐of‐package food labelling and applying 8% tax on non‐essential energy‐dense food and one Mexican peso per litre tax on SSB [56]. The tax was enacted in 2014 as an amendment to the existing Special Tax on Production and Services (IEPS), which came into effect in 1980 but applied only to alcohol and tobacco until then. This tax remains in force, increasing by 4.3% in 2024.

#### 3.2.3 The Politics

Mexico's Federal Government is organised around a president elected every six years and a bicameral parliament elected every three years. The soda tax was included in the strategic Presidential National Development Plan (NDP 2013–2018) as part of a set of measures to reduce the prevalence of obesity under the newly elected President from the centre of the political spectrum, Institutional Revolutionary Party (Partido Revolucionario Institucional). The operational delivery of the plan was led by the Ministry of Health and the Ministry of Finance. The Ministry for Health was not supportive of the tax and suggested that raising awareness and educating the population would be more appropriate interventions [61]. The original proposed 20% tax rate was changed to 1 Mexican peso per litre. This new tax rate took academics and the civil society by surprise, and the exact reasons for this remain unclear; however, it was speculated that this was the result of the influence of the food and beverage industry (F&BI) [61]. Despite the government suggesting that the SSB tax was developed through a multi‐stakeholder approach, this was disputed by the civil society, which pointed out the disproportionate influence of the F&BI on the policy development.

Key to success was the mobilisation of NGOs that developed a major social communication strategy. This was facilitated by a $16.5 million donation from the Bloomberg Philanthropies Foundation to carry out campaigns and strategic communication interventions [62]. This donation was crucial to counterbalance the financial power of Mexico's F&BI [63]. Relevant messages were shared on billboards and posters in subway stations, busy streets and avenues and paid inserts were placed in the main newspapers. Members of NGOs and national research institutes participated in radio and television programmes and featured in the written press. In addition, academics and representatives of the United Nations, NGOs and research institutes attended technical meetings in the Senate to review the proposal and support the development of an evidence‐based strategy [59].

At the same time, the F&BI formed a united front against the tax, with significant activism in the media. One strategy was to create uncertainty, especially in financial matters, by putting forward opinion leaders, medical and nutrition professionals, to argue against the tax, highlighting its potential economic impact on job losses and revenue. The industrial sector also engaged in intense and, at times, inappropriate lobbying in Congress, the state secretariats and other regulatory entities. There is evidence that various relationships between the F&BI and health organisations, the Mexican Federation of Diabetes and other NGOs were not publicly disclosed during the SSB tax discussions [59, 64]. For example, the Health Secretary was previously the chief executive of the Mexican Foundation for Health (FUNSALUD), a research charity sponsored by Nestle [61]. The Latin American Federation of Diabetes positioned itself against the soda tax, without disclosing the funding received from Coca‐Cola [61].

### 3.3 Junk Food Tax in Colombia

The complex dynamics between private industry and politics mean that implementing change often requires a gradual approach and some degree of flexibility as illustrated by the development and implementation of the “junk food tax” in Colombia.

#### 3.3.1 The Problem

Between 2005 and 2010, there was a 10% increase in overweight prevalence, as shown by the 2010 Colombian National Nutrition Survey [65]. According to the same survey, 81.2% of Colombians consumed SSB, and 13% of deaths from diabetes could be related to that consumption. Estimates from the Ministry of Health specified that the tax would have had a significant impact on SSB consumption, and approximately 220 000 million Colombian pesos (approx. US $75300000) could have been saved per year in health care expenses for patients with diabetes [65].

#### 3.3.2 The Policy

Before the current health tax (2023), there was an attempt to implement a 20% tax on SSB in Colombia in 2016, led by the Health Minister representing a centre‐right government. That initiative was supported by members of the civil society, such as Educar Consumidores (an NGO that works on consumer issues that affect human and environmental health). Starting in 2015, Educar Consumidores led a public health campaign on national television channels funded by Bloomberg Philanthropies, which included informative videos related to the consumption of SSB and the implementation of the tax. This campaign was sued on misleading advertising grounds by Gaseosas Postobón S.A., one of the main SSB producers in Colombia, and the videos were removed. Although the 2016 tax proposal was not approved in Congress, it was the first step for civil society to become more visible in their advocacy efforts and the start of a “movement” that will later be successful.

As part of the industry's response to its damaged reputation, in 2017, Gaseosas Postobón S.A. began a strategy focussed on promoting its supposedly “super nutritious” new product called Kufu, which contained 13 g of sugar per bottle. Kufu was distributed to children in La Guajira, one of the poorest areas in Colombia, where levels of child malnutrition are very high. NGOs warned about the harmful effects of the beverage on health, and the company immediately withdrew it from the market without any legal consequences [66].

Later, in 2021, as a result of the COVID‐19 pandemic and the government's proposal of an arbitrary tax reform aimed to impose additional taxes on basic foods, there was an unprecedented social outbreak in Colombia. One of the indirect achievements was the approval of the so‐called “‘Junk Food Law”, focussed on front labelling of ultra‐processed foods (UPF) and SSB. At first, the labelling did not comply with the scientific evidence and regulations, so RedPapaz (another NGO) sued the government for the way the law was being implemented, and they were successful. As a consequence, modifications supported by scientific evidence were made, including no illustrations, an octagonal shape to capture the consumer's attention, and black colour, which conveys a feeling of unhealthy [67].

#### 3.3.3 The Politics

The next presidential and legislative elections took place in 2022, and the left‐wing political party won the presidential elections. During the political campaign, the elected president expressed interest in the implementation of the “health tax”. In the same year, La Liga Contra el Silencio (a group of independent journalists) uncovered that the UPF and SSB industry funded several campaigns of elected congressmen, which meant a conflict of interest for decision‐makers [68, 69]. Fortunately, and despite the political power of the industry, the law was approved by both the Congress and the Constitutional Court, making the Colombian health tax on UPF and SSB a reality. During the negotiation process, the government held a debate and made certain tax parameters a little more flexible, for example, the starting date for the tax to be collected. Given the strong and powerful economic interests of the industry, without this compromise, the tax could not have been implemented [70].

As of November 2023, the health tax came into effect. The tax is directly proportional to the added content of sugars, sodium and saturated fats. In addition, the tax will gradually increase from 10% in 2023 to 20% in 2025 [70]. It is very important to highlight the role played by NGOs and universities in this process, which, combined with a change in the national political landscape, made possible the Colombian junk food tax. The coming years are key in terms of evaluating the impact of the tax on the health of the Colombian population.

### 3.4 Minimum Unit Pricing for Alcohol in Scotland

A number of studies have explored in great detail the barriers and facilitators in developing and implementing the alcohol minimum unit pricing (MUP) policy in Scotland and the role of evidence, political climate and various interest groups in influencing and shaping policy development [12, 24, 71, 72, 73].

#### 3.4.1 The Problem

Alcohol consumption is one of the leading causes of premature deaths worldwide and is a significant risk factor for several long‐term conditions, including oral diseases [74, 75]. Worldwide, 4.1% of all new cancer cases have been attributed to alcohol consumption [76].

In the UK, although alcohol prices increased in line with inflation, the increased living standards meant that alcohol was 74% more affordable in 2020 than it was in 1987 [77]. The increased affordability was linked with an increase in alcohol consumption as well as in the prevalence of liver disease. Between the 1980s and early 2000, mortality associated with liver disease increased by two‐thirds in England and Wales, and it doubled in Scotland, making this the highest increase in Western Europe [78]. Associated health inequalities exist within Scotland, with chronic liver disease rates being nearly four times higher in the most socio‐economically deprived areas compared to the most affluent areas [79].

In the context of MUP development, the problem was evidenced by robust epidemiological data presenting the burden of alcohol‐related harms and how Scotland was an outlier among other similar countries [78]. It is worth noting that beyond the statistics, there is ample evidence supporting the role of stories in influencing policies [80]. An interviewee in a study exploring the development of MUP in Scotland mentioned how it worried them that alcohol could be cheaper than a bottle of water, a powerful point that was also included in the centre‐left Scottish National Party (SNP) manifesto in 2007 [24, 81].

#### 3.4.2 The Policy

In 2002, the Scottish Labour (centre‐left)–Liberal Democrat (centrist to centre‐left) coalition introduced a “Plan for Action on Alcohol Problems”. This was an innovative policy, but still focussed mainly on “problem drinkers” and on individual responsibility. This was followed by the Licensing Act (2005), which introduced five licensing objectives, including one centred around the protection of public health [82].

In 2007, a minority government led by the Scottish National Party (SNP) (centre‐left) came into power, and at the same time, the Scottish Medical Royal Colleges and Faculties established a new advocacy group called the Scottish Health Action on Alcohol Problems (SHAAP). The group was operating independently but was funded by the Scottish Government with the aim of raising awareness about alcohol‐related harms and promoting solutions based on the best available evidence [24, 83]. The recommendations of SHAAP were focussed on whole population approaches rather than only “problem drinkers” and advocated for the introduction of MUP. Following the elections of 2011, the SNP gained an overall majority of seats in the Scottish Parliament, and the next year, it passed the Alcohol (Minimum Pricing) Bill [24, 82, 84]. The Scottish Whisky Association (SWA) challenged the legality of MUP. Both the Scottish Courts and the UK Supreme Court ruled in favour of the Scottish Government to implement this policy. The SWA appealed this decision at the European Court of Justice (ECJ), which supported the SWA appeal but left the final decision on the matter to the domestic courts. It is also worth noting that, once the case was brought in front of the ECJ, the UK Government (then an EU Member State) had formal standing in front of the Court and not the Scottish Government, creating additional levels of interdependency for the policy between the parties. This further demonstrates the ability of transnational corporations to operate in a co‐ordinated way across different levels of governance and jurisdictions in order to pursue their goals, and the level of effort required by those trying to oppose the influence of industry on policy changes [73].

Due to the numerous legal challenges and appeals, the implementation of MUP was delayed by six years [73]. This delay may have had significant consequences beyond just the time element. As evidence suggests, the implementation of MUP resulted in a 13.4% reduction in deaths and a 4.1% reduction in hospitalisations wholly attributable to alcohol; therefore, we can assume that there is a quantifiable number of deaths and hospitalisations that could have been avoided if MUP had been implemented sooner [76]. Here, for the first time, we provide an estimate of the impact of delaying the implementation of MUP. If the annual impact of the policy is a reduction of 156 deaths and a reduction of 411 hospital admissions wholly attributable to alcohol each year, and assuming the strength of the intervention and associated benefits were consistent with the evaluation evidence, the extrapolation from this would mean that approximately 936 deaths and 2466 hospitalisations could have been avoided [76]. Assuming the average cost of hospitalisations is £2971/case, the hospitalisations wholly attributable to alcohol in the six‐year delay represented a cost of more than £7 million for the NHS Scotland [85].

Policy was developed offering a feasible solution to the problem. This was undertaken by the multidisciplinary group of experts involved in the SHAAP, who examined the totality of available evidence and made a number of recommendations, while also advocating for the introduction of MUP. The effectiveness of SHAAP's communication strategies and style was crucial in framing the conversation with the civil service and politicians, condensing the evidence and presenting it in accessible language easily translatable into policy solutions [86]. SHAAP was working closely with public health organisations, which were in close relationship with the Scottish Government, to deliver clear, concise and consistent messages to policymakers directly and through the media to the wider population [86]. The Government, in turn, was keen to rely on the “in‐house” expertise provided by public health organisations to inform the development of policies [86]. It is important to note the role that “policy entrepreneurs” played in this process. These are influential people who can champion a cause and use their political weight to shape policy development. In the case of MUP among others, one key player was Nicola Sturgeon, the then Health Minister and Deputy First Minister, who later became Scottish First Minister [24].

#### 3.4.3 The Politics

On one hand, the political climate was facilitated by the new government that had a view which was more focused on tackling the issue at hand and diverged from working through a partnership approach with industry—who were steadfastly opposed to price policy reform [11]. Additionally, developing a Scotland‐specific policy to tackle health problems in Scotland was perceived favourably for the centre‐left party of government (the SNP). The initial media coverage of the topic framed the issue around a minority of youth “binge drinkers” masking the true magnitude of the problem at the population level [87]. Over time, two polarised coalitions were formed: the proponents and the opponents of MUP. The proponents were mostly health advocacy groups, charities, political parties, and academic institutions, while the opponents were the main alcohol manufacturers and economic think‐tanks, along with opposition political parties [11]. The proponents shared through the media concepts about the need for government intervention to reduce alcohol consumption, the role of government in limiting commercial interests in order to protect public health and the need for MUP. At the same time, the opponents were promoting themes such as falling/stabilising trends for alcohol‐related harms, unnecessary government intervention, unfairness of MUP and other similar concepts meant to create confusion and uncertainty [11].

### 3.5 Inclusion of Boys in the HPV Vaccination Programme in the UK

Public health organisations advocating for preventive interventions need to consider the landscape in which policy decisions are made and be flexible in providing different types of evidence to strengthen the argument. In the case of HPV vaccination, this meant considering not just cost‐effectiveness but also gender equality issues.

#### 3.5.1 The Problem

In recent decades, incidence rates of oropharyngeal cancers have been among the most rapidly rising cancers across Europe, trends driven largely by human papillomavirus (HPV) [88, 89]. The prospect of primary prevention through HPV vaccination, originally designed to prevent cervical cancer in women, was being proposed because of strengthening new evidence on the efficacy of HPV vaccination in preventing HPV‐driven non‐cervical cancers [90]. However, many countries (including the UK) implemented a female‐only vaccination programme, maintaining that males would be protected via herd immunity, and with men who have sex with men offered targeted vaccination programmes [91]. Internationally, 47 countries had implemented a gender‐neutral HPV vaccination programme by 2022 [92].

#### 3.5.2 The Policy

Since 2013, the Joint Committee on Vaccination and Immunisation (JCVI), which brings together scientists, public health professionals and policymakers from across the four UK nations, has been considering evidence on whether the HPV vaccination programme should become gender neutral.

In 2017, the initial cost‐effectiveness modelling provided to JCVI predicted that extending the HPV programme to adolescent boys would not be a cost‐effective use of health service resources in the UK [93]. Later in 2018, JCVI decided to include additional analyses before concluding its advice [93]. The JCVI modified its standard criteria for cost‐effectiveness evaluation and considered that a lower discount rate (1.5%) could be appropriate to better take into account the longer term impact of HPV vaccination in cancer prevention, and that under the combined girls' and boys' programme compared to no vaccination, gender‐neutral HPV vaccination would be cost‐effective [94]. By 2019/2020, the UK Westminster and devolved nation governments took the decision based on evidence and equality legislation to extend the HPV vaccination programme to include boys across the UK.

#### 3.5.3 The Politics

This work took place under a conservative (centre‐right to right‐wing) UK Government. Throughout the deliberations of the JCVI, there was a sustained wide and loud advocacy campaign to extend the HPV vaccination to include boys. Under the collaborative umbrella of HPV Action, over 50 professional and patient organisations, including oral health and dental organisations, had signed up in favour of this [91]. Early successes included media stories, parliamentary motions and questions and cross‐party politicians' support. Legal proceedings were also taken against the UK Government on the grounds of gender discrimination under the equality legislation. The JCVI recommended an equality analysis delivered by the UK Department of Health and Social Care (DHSC) to support the potential extension of HPV vaccination to adolescent boys. The DHSC review concluded that vaccinating boys provided an opportunity to advance equality, providing boys with direct protection and would reduce the overall responsibility of girls in protecting the population's health. It also reinforced the UK's commitment to a world‐class vaccination programme as well as to cancer prevention and improved sexual health [95]. One of the main enabling factors for changing the JCVI decision was based on shifting the framing of the problem from an economic and effectiveness evidence standpoint to one, which focussed on equality. With the peak incidence of oropharyngeal cancers being at 50–69 years, the prospects of the impact of the vaccine on oropharyngeal cancer are likely some decades away [89]. In the meantime, alternative secondary prevention, early detection programmes, e.g., improving access to dental care services for opportunistic oral examinations, will be needed. With still less than two‐thirds of the world's countries implementing HPV vaccination programmes and most of them (70%) including females only, there are policy‐influencing lessons to be drawn [96].

### 3.6 Tobacco Control Legislation in Aotearoa New Zealand

Despite implementing ambitious legislation to reduce health inequalities between the indigenous populations and other ethnicities, without continued political support, these laws can be easily repealed, and any progress reversed.

#### 3.6.1 The Problem

Despite a decrease in smoking prevalence in most high‐income countries, indigenous populations in countries with a colonial history are disproportionately affected by the associated morbidity and mortality [97]. As such, health inequities in Aotearoa New Zealand are persistent [97]. The attribution of inequity to surface causes (i.e., health practices, psychosocial resources or health system access) or social status (i.e., socio‐economic position or ethnicity) ignores further upstream drivers of structural inequity, i.e., key principles such as the colonial basis of dominant culture, economic structures and political and legal systems [98]. Specifically, in Aotearoa New Zealand, Māori people have been politically, economically and socially undermined, leading to lower income and life expectancy, poorer education, and stigmatisation within health care [97].

A 2010 Māori Affairs Committee inquiry into the tobacco industry in Aotearoa and the consequences of tobacco use for Māori found that, while overall smoking rates in Aotearoa New Zealand were decreasing, rates among Māori and Pacific peoples were actually rising [99]. In addition, Māori women, in particular, had among the highest global rates of lung cancer [99]. The inquiry highlighted the cultural cost of tobacco on Māori and emphasised the importance of eliminating tobacco to preserve Māori culture for younger generations, ultimately setting the stage for New Zealand's goal of becoming smokefree by 2025. Furthermore, in 2021, almost 20% of Māori and Pasifika populations smoked daily compared with 7% of European/Other ethnicities in Aotearoa New Zealand [100]. At the same time, Māori mortality rates are at least twice as high as the non‐Māori population [101]. Inequalities in tobacco‐related health outcomes can be attributed to several factors, including the lasting effects of colonisation and the tobacco industry's exploitation of the social and financial vulnerabilities of indigenous peoples, actively promoting and encouraging the use of smoked tobacco products among this target audience [100, 102].

#### 3.6.2 The Policy

For several decades, Aotearoa New Zealand has maintained a relatively strict regulatory approach towards tobacco [103]. In December 2022, New Zealand enacted world‐leading tobacco control legislation aimed at leading the nation towards a “smokefree” future by 2025, a future where the smoking prevalence falls below 5% across all population groups. To achieve this goal, revolutionary measures were needed [103]. The new legislation was meant to reduce the number of tobacco retailers by 90%, denicotinisation of retail tobacco and progressively increase the legal age for purchasing tobacco (which prohibits the sale of tobacco products to individuals born on or after January 1, 2009). The modelling behind these measures estimated that their cumulative effect would result in NZ$1.3bn (US $761 m) savings in healthcare costs, prevention of over 8000 deaths over 20 years and narrowing of Māori health inequalities for both men and women [100, 104].

#### 3.6.3 The Politics

Māori leaders called for a return to the country's original tobacco‐free status as tobacco was originally introduced by European colonists and its use was perceived as a manifestation of colonialism [104]. In 2011, the National Party (centre‐right) led government adopted a smoke‐free goal for 2025. Later on, the Labour (centre‐left) government adopted an ambitious and comprehensive set of legislative measures with strong community support, which came into force in early 2023 [104]. However, the tobacco industry represented by British American Tobacco New Zealand (BATNZ) and Imperial Brands Australasia (IBA) strongly opposed this policy by seeking alliances with retailers and initiating public petitions. When these strategies failed, BATNZ and IBA initiated a more aggressive strategy under the umbrella “Save our Stores” (SOS) campaign, claiming that the policy would lead to a fatalistic future where the retail industry would collapse, leading to unemployment, increased cost of living, while crime and black markets would flourish [105]. The intention of these metanarratives was to exaggerate the policy's aims, ignoring any potential benefits and portraying government intervention as “nanny state”. Additionally, these strategies aimed to undermine public support for the policy and pave the way for its reversal.

Following the 2023 parliamentary elections, the National Party (centre‐right) won most seats and entered into a coalition government with the right‐wing libertarian ACT party and the right‐wing populist New Zealand First party. The internal negotiations led to the repeal of the new tobacco control legislation in February 2024, along with other acts like repealing the Māori Health Authority [97]. The new finance minister suggested that revenue generated from tobacco taxation would support the economy. This argument, however, ignores the health and social care costs, loss of productivity and deaths caused by smoking [104]. The reversal of this health policy is anticipated to result in thousands of lives lost and widen life expectancy gaps between indigenous and non‐indigenous populations. This decision, driven by political ideology and interference from the tobacco industry, has not only impeded Aotearoa New Zealand's progress, but also weakened global efforts in tobacco control [103].

**TABLE 1:** Summary findings from the case studies using the three dimensions of Kingdon's framework.

Country	Scotland	Mexico	Colombia	Scotland	United Kingdom	Aotearoa New Zealand
Case study	Childsmile	Tax on sugar‐sweetened beverages	Junk food tax	Minimum Unit Pricing (MUP) for alcohol	Inclusion of boys in HPV vaccination programme	Tobacco control legislation
Problem	In the early 2000s, among the highest prevalence of dental caries in children in Western Europe Wide socioeconomic inequalities Opposition to community water fluoridation	High prevalence of obesity & associated conditions, including caries Among the highest levels of soft drinks consumption across 75 countries & the second largest consumer of ultra‐processed foods (UPF) and beverages in Latin America	High prevalence of obesity & associated conditions, including dental caries Modelling shows the economic benefit of the tax in terms of healthcare savings	Increased affordability and consumption of alcohol are linked with increased morbidity/mortality Scotland saw the highest increase in alcohol‐related harms in Western Europe	HPV‐driven increased incidence of oropharyngeal cancers among highest in Europe The initial approach was around female vaccinations only; males were protected through “herd immunity”	High prevalence of smoking and associated morbidity/mortality in Māori and Pasifika populations compared with other ethnicities
Policy	Regional pilots merged into a national programme aimed at reducing both caries prevalence and inequalities Multilevel intervention based on proportionate universalism Embedded monitoring and evaluation showing reduction in caries in 5‐year‐old children from 55% in 2003 to 27% in 2024; and in 11‐year‐olds from 47% in 2005 to 18% in 2023	Policy window: the new government needed to increase revenues to the treasury and planned to increase taxes on all foods, medicines and non‐alcoholic drinks; failed to progress because it lacked support, but it allowed framing the issue for a “health tax” Intersectoral support across federal government, congress, academia, non‐governmental organisations (both local and international), and international health agencies	Early attempts to introduce the tax have failed, but managed to bring the issue into mainstream public and political awareness The COVID‐19 pandemic opened a policy window as government proposed a tax reform to increase revenues	Initial attempts focussed on individual “problem drinkers” Scottish Health Action on Alcohol Problems (SHAAP), bringing together civil society, academia and professional organisations SHAAP recommendations on population‐level approaches supporting MUP. New government passed MUP legislation but faced legal challenges by alcohol industry, delaying implementation for six years. Role of “policy entrepreneurs” championing for the cause	From a cost‐effectiveness standpoint, vaccinating boys was not considered cost‐effective	New ambitious legislation, based on modelling to improve health outcomes and reduce inequalities
Politics	Continued support over almost 20 years under successive governments Started under Labour‐Liberal Democrat coalition; sustained under successive SNP governments, all in the centre‐left of the political spectrum Evidence from monitoring and evaluation informing government policy Continued strategic communication with stakeholders across all levels: government; regional directors for health and education; dental practices and schools	Initial opposition from the Ministry of Health, but support from the National Institute of Public Health Institutional Revolutionary Party (Partido Revolucionario Institucional) (centre). Originally proposed 20% tax changed to 1 Mexican peso/l potentially as a result of industry influence. Strong efforts by the food and beverage industry (F&BI) aimed at creating uncertainty Partnership between civil society, academia, and international organisations to counterbalance private industry narrative and seek public support	Strong industry funding for various political campaigns during the elections New (centre‐right) government passed legislation on a progressive tax on ultra‐processed foods (UPF) and sugar‐sweetened beverages (SSB) introduced gradually over time Role of NGOs, academia and changing political landscape in favour of the tax	Supported by centre‐left government. Favourable political landscape for a Scotland‐specific policy Strong messaging using robust data and modelling moving the focus from individual “binge drinkers” to whole population approaches	Adopted by centre‐right to right‐wing government. Strong advocacy from patient and professional organisations Legal proceedings against government on the grounds of gender‐based discrimination Government‐revised decision based on equity and equality rather than just health economics	Support from Māori leaders as smoking is seen as a colonial influence Started by centre‐right government, strengthened by a different centre‐left government and then abolished by centre‐right, right‐wing libertarian populist coalition government Strong opposition from the tobacco industry suggested that the policy would lead to the collapse of the retail industry, economic instability and chaos Negotiations within the new coalition government led to repeal of the legislation

## 4 Key Lessons From Case Studies for Policy Development and Implementation

We identified a number of shared lessons, barriers and facilitators for policy development and implementation arising from the six case studies.

### 4.1 Barriers

1. Misinformation and evidential landscaping used by some industries to create confusion and minimise the impact of harmful products on health, encourage interventions which focus on personal responsibility alone rather than government intervention that could address the broader social, environmental and political determinants.
2. Use of legal challenges with the aim of delaying policy implementation.
3. Lobbying politicians, academia and civil organisations against the implementation of public health policies either directly by the industry or indirectly through industry‐funded organisations.
4. Media campaigns aimed at creating confusion and influencing public opinion. These often include the distribution of promotional products free of charge.
5. “Libertarian” ideology portrays any government intervention as “nanny state” and a loss of freedom.
6. Lack of transparency from stakeholders in actively disclose conflicts of interest.

### 4.2 Facilitators

1. Robust and timely scientific evidence in support of the policy. It is good practice to include options appraisal and cost‐effectiveness modelling, presented in an accessible manner but without being oversimplified.
2. Identification of key decision‐makers to optimise and streamline the advocacy process.
3. Having a dynamic plan of action to accommodate changes in the political landscape.
4. Public health policy can be advanced under governments of any political orientation through the strategic use of evidence and advocacy.
5. Cross‐party support that enables continuity of political support and funding even after government changes.
6. Opposing “libertarian” ideology by highlighting individuals' right for a long healthy life, free from disability and debilitating conditions, and free from addictive substances like nicotine.
7. Strong civil service that facilitates operational support and delivery.
8. Monitoring and evaluation for quality improvement and evidence of effectiveness.
9. Intersectoral collaboration between healthcare and public health organisations, academia and civil society.

These barriers and facilitators have been essential for policy development and implementation for the six case studies presented in this paper. At the same time, it is important to recognise the role of complexity and intentionality in exploring these drivers.

In terms of complexity, policy development and implementation are operating under complex dynamic systems rather than linear cause‐and‐effect models. A binary categorisation might oversimplify the reality and miss the deeper structural and contextual factors that influence these policies, including the macroeconomic and political models in which the policies are developed. It is important to acknowledge the influence of local context and to move beyond simplistic classifications by examining how the barriers and facilitators interact within different policy environments, contributing to a more nuanced understanding of the policy processes [106].

In terms of intentionality, many of the mechanisms listed as barriers or facilitators are not inherently positive or negative in themselves. Rather, they are tools that different actors may use depending on their intentions to either support or oppose policy change. For example, public health advocates have successfully used media campaigns to shift public opinion in favour of tobacco control and sugar‐sweetened beverage taxation, while industries have used similar strategies to undermine such initiatives. Similarly, legal challenges can be used to delay the implementation of evidence‐based policies, but they can also be deployed to hold governments to account for implementing public health measures against corporate interests. Other tools, such as lobbying, framing of public narratives and coalition building, similarly depend on how they are being used and by whom. Recognising this complexity provides a more realistic understanding of the policy environment, where the same tools can serve very different purposes depending on the intentions of those wielding them in these complex environments.

### 4.3 Strengths and Limitations

The case studies included in this review cover a broad spectrum of policies with relevance to population oral health. These cases originated from a number of different countries with different political traditions and systems and welfare state regimes [107]. The authors that led on each case are based in the countries described in the cases, bringing a personal perspective on the wider social, political, and cultural context in which the policies were developed. While this strengthens the contextual depth of the analysis, differences in the interpretation of political events across cases should be acknowledged as a potential source of bias.

This review used a multiple case study approach, applying Kingdon's Multiple Streams Model to analyse the policy development processes for policies with relevance to population oral health. This model provides a robust framework for understanding how policies were developed, but it does not fully capture the long‐term implementation challenges of public health policies. We used this model because it captures most comprehensively the political‐policy context and the role of policy entrepreneurs in influencing change during windows of opportunity, making it highly relevant to our case studies. Alternative theories, such as the Advocacy Coalition Framework and the Equilibrium Theory, provide important perspectives on researching policy processes but are less suited for the focus of this study. The Advocacy Coalition Framework focusses on long‐term policy stability rather than short‐term openings for change, while the Punctuated Equilibrium Theory is better suited for explaining abrupt policy shifts rather than ongoing policy evolution [108, 109].

Although the primary focus of this paper was policy development and agenda‐setting, we have also incorporated elements from Implementation Science by extracting the main barriers and facilitators from across the case studies. This allowed us to highlight the key factors that may influence policy development as well as, to some degree, implementation and sustainability. However, future research might benefit from a more in‐depth exploration of Implementation Science, particularly by examining the complexities surrounding barriers and facilitators and the interplay between these factors [110].

Policy decisions do not occur in isolation but are shaped by the broader macroeconomic and political contexts in which they are located. The welfare state regimes provide an important framework for understanding how different countries approach social policies in general, and public health in particular, including oral health policies. Oral health inequalities persist across all welfare state regimes and, contrary to expectations, they are not necessarily smaller in the more egalitarian welfare regimes (e.g., the Scandinavian regime) [107]. However, welfare state models are not static categories. The UK, for example, while historically classified as a liberal welfare state, has undergone significant transformations in its welfare system due to political and economic pressures, including recent austerity measures and market‐oriented reforms. These dynamics have been highlighted by more recent scholars describing how austerity measures and reductions in state intervention can have broader public health and political consequences, creating a shift towards more populist far‐right political parties [111, 112]. Recognising these macro‐political forces provides a more comprehensive understanding of the barriers and facilitators in policy development, ensuring that political decisions are contextualised within evolving welfare systems.

### 4.4 Where Next? The Way Forward

An important priority for the public health community is the need to become more politically astute to better influence the development of policies impacting health [113, 114]. As seen in the case studies presented in this paper, it is important to be able to work with all parties across the political spectrum and provide objective, evidence‐based professional advice to politicians and policymakers. The dynamics between the private sector and political actors are complex and cannot be oversimplified as “good” or “bad”. However, it is important to examine each actor's primary obligations: the private sector's obligations to maximise profits for shareholders and politicians' duty towards the population and political party they represent [114]. It is also worth noting the structural challenges around the timing of political cycles, which could be hindering ambitious public health interventions [10, 115, 116]. In most countries, local, regional and national politicians are re‐elected every 4–6 years. Public health interventions require long‐term commitment, often spanning across successive governments, in order to produce the desired effects at the population level, like the example of Childsmile in Scotland. In this context, it can be tempting for politicians to seek “quick wins” to convince the electorate about their successes in office and seek re‐election, often ignoring the long‐term effects and trade‐offs associated with these “quick wins” [117]. Balancing the need to respond to political demands from politicians while still achieving implementation of evidence‐based health interventions requires cycles of negotiation, some degree of flexibility and delimited commitments among different stakeholders as it was the case of the junk food tax implementation in Colombia and the decision to vaccinate boys as part of the HPV vaccination programme in the UK.

The conflicting economic interests of certain industries and some civil organisations in the process of development, implementation and evaluation of health policies are unavoidable and have been documented previously, including the case studies presented in this review [118]. The approach used by some industry actors includes disputing objective scientific evidence while promoting a biased evidence base that aligns with their policy goals and creates confusion among policymakers about responsibility for harms caused by certain products [119]. For instance, despite objective and measurable evidence of MUP reducing alcohol‐related harms and despite being endorsed by all member states of the World Health Organisation, there are few countries where MUP is currently implemented. Industry has been downplaying any negative trends in alcohol‐related harms and attributed any positive trends to industry‐supported interventions. An example of this can be seen through a recent parliamentary oral evidence session on alcohol harms organised by the Health Select Committee of the UK Parliament [120]. The first session included four organisations, all of which were funded by the alcohol industry, yet no conflicts of interest were declared at the opening of the session. The aim of the parliamentary committee was to gather evidence on preventing alcohol‐related harms, yet the evidence provided by the panellists avoided the WHO recommendations on evidence‐based interventions through policies targeting affordability. Instead, the panellists recommended interventions with no evidence base, such as public information campaigns. Similar strategies have been adopted by industry actors in influencing the development of the SSB tax in Mexico [61]. These types of tactics need to be challenged by the public health community [119]. Establishing a framework for evaluation of evidence, including validated scientific evidence and mandatory disclosure of conflicts of interest beforehand, can be the starting point to counteract the biased evidence industry could use in policy evaluations, as in the case of the tobacco control legislation in New Zealand [103, 121].

The literature around the political determinants of oral health is emerging; however, there is ample literature on the policy development processes in similar areas such as other non‐communicable diseases or diet and obesity [122]. Our findings are consistent with the evidence found in these areas [123, 124].

The contribution of the academic and public health community to the development, implementation and evaluation of oral health policies needs to proactively engage with the political landscape [125, 126, 127]. Important ways forward for reaching these goals include:

1. Dissemination of research cannot be limited to peer‐reviewed publications and scientific audiences. Researchers should not limit communications to academic spaces. Joint research evidence needs to be tailored to reach a broader public from practitioners to policymakers and civil society. Thus, communication of research evidence for policymaking purposes needs to be clear, accessible, usable and timely [124].
2. Training dental public health workforce with the skills required to work across multidimensional stakeholder partnerships at local, national and international levels. This includes system leadership, advocacy and expert communication skills, which allow understanding and better engagement with policymakers [128].
3. Oral health research needs to increasingly focus on the needs of local and national governments and civil society. Research needs to shift from highlighting only the problems towards focussing on potential solutions, as this will make it policy relevant and can have a real‐world impact on population oral health and reducing inequalities.
4. The underlying principles for research need to drive the integration of scientific and political values: sensitivity, being respectful of the context, inclusiveness, identifying all actors/stakeholders needed to be involved in the different stages of the policy programmes, transparency when controlling access to information, deliberation and consensus of the problem while assuring reciprocity and multidisciplinary contributions, considering all angles and legitimacy at all stages of the process [129]. Creating more opportunities to jointly develop a Science Diplomacy approach for oral health will connect the scientific evidence, the intersectoral deliberation of the problem and incorporation of the politics and the power structure (directly defining how to develop, manage and maintain health policies/programmes) [115].
5. Academic institutions could work more directly with the health/science ministries, governmental and private research funding agencies and NGOs to develop the road map to identify research priorities and funding strategies. This will include the need for more oral health policy‐focussed research collaborations, both locally and globally. An example of such strategies includes the CORE (Community‐Focussed Oral‐Health Research for Equity) Programme, a four‐year research programme funded by the UK National Institute for Health and Care Research (NIHR) to address the neglect of oral diseases with a policy focus in four middle‐income countries—Colombia, Kenya, India and Brazil [130].
6. Research on the advocacy process itself ought to be part of the research agenda in order to not only keep pace with but outperform the latest methodological innovations from the private sector, while also adopting a strategic approach that effectively influences policymakers and drives change at the population level [115].

## 5 Conclusion

In an increasingly fast‐paced and interconnected world, with national and international politics influenced by powerful commercial and media interests, public health professionals and those working in oral health/dentistry leadership roles, have a professional and ethical responsibility to work with governments, academics and non‐governmental organisations to provide balance in the political decision‐making process in the interests of population (oral) health. While presenting robust research evidence to policymakers, honest conversations need to consider the trade‐offs for action as well as for non‐action. Government interventions promoting public health measures are not necessarily in a zero‐sum game against the economy, as economic prosperity is not possible without a healthy population [131, 132, 133]. Ending with the wise words of Prof Sir Michael Marmot and Dr. Venkatapuram, “Now is the time to embed the idea and understanding that health, health equity, and a good and just society are interlinked. A vision for a better world on the other side of all of this current chaos will undoubtedly emerge sooner or later. Dental public health and public health more generally must aim to ensure health justice is at the centre of it” [134] alongside our conclusion and call for action to engage with the politics, with the politicians—the political determinants—to shape policies to address oral health inequalities.

## Disclaimer

Where authors are identified as personnel of the International Agency for Research on Cancer/World Health Organisation, the authors alone are responsible for the views expressed in this article and they do not necessarily represent the decisions, policy or views of the International Agency for Research on Cancer/World Health Organisation. C.G.‐H., M.K.N.‐R. and D.I.C. are part of the CORE (Community‐Focussed Oral‐Health Research for Equity) project, a research programme funded by the UK National Institute for Health and Care Research (NIHR). S.R.B. currently has two Haleon (GSK) funded research projects and has received a number of grants from Haleon (GSK) over the previous years. G.T. contributes to research projects, not related to this paper, that have received funding from the UK National Institute for Health and Care Research (NIHR) and from the UK Department of Health and Social Care. D.I.C. and S.T.S. received funding from the Scottish Government to monitor and evaluate the Childsmile programme. D.I.C. is the chair of the Socialist Health Association Scotland.

## Conflicts of Interest

The authors declare no conflicts of interest.

## Data Availability

Data sharing is not applicable to this article as no new data were created or analyzed in this study.
